# Electrical Stimulation of Inner Retinal Neurons in Wild-Type and Retinally Degenerate (*rd/rd*) Mice

**DOI:** 10.1371/journal.pone.0068882

**Published:** 2013-07-11

**Authors:** Morven A. Cameron, Gregg J. Suaning, Nigel H. Lovell, John W. Morley

**Affiliations:** 1 School of Medicine, the University of Western Sydney, Campbelltown, New South Wales, Australia; 2 Graduate School of Biomedical Engineering, the University of New South Wales, University of New South Wales, Sydney, New South Wales, Australia; University of Houston, United States of America

## Abstract

Electrical stimulation of the retina following photoreceptor degeneration in diseases such as retinitis pigmentosa and age-related macular degeneration has become a promising therapeutic strategy for the restoration of vision. Many retinal neurons remain functional following photoreceptor degeneration; however, the responses of the different classes of cells to electrical stimuli have not been fully investigated. Using whole-cell patch clamp electrophysiology in retinal slices we investigated the response to electrical stimulation of cells of the inner nuclear layer (INL), pre-synaptic to retinal ganglion cells, in wild-type and retinally degenerate (*rd/rd*) mice. The responses of these cells to electrical stimulation were extremely varied, with both extrinsic and intrinsic evoked responses observed. Further examination of the intrinsically evoked responses revealed direct activation of both voltage-gated Na^+^ channels and K^+^ channels. The expression of these channels, which is particularly varied between INL cells, and the stimulus intensity, appears to dictate the polarity of the eventual response. Retinally degenerate animals showed similar responses to electrical stimulation of the retina to those of the wild-type, but the relative representation of each response type differed. The most striking difference between genotypes was the existence of a large amplitude oscillation in the majority of INL cells in *rd/rd* mice (as previously reported) that impacted on the signal to noise ratio following electrical stimulation. This confounding oscillation may significantly reduce the efficacy of electrical stimulation of the degenerate retina, and a greater understanding of its origin will potentially enable it to be dampened or eliminated.

## Introduction

Current strategies to restore vision to those suffering from retinal degeneration are varied, ranging from re-growth of photoreceptors from stem cells, to using electrical stimulation to activate the remaining cells in the retina. To be of use clinically, all these strategies, whether using purely biological tools or complex electrical engineering, must interact with the degenerate retina of the human eye. It is therefore essential that the physiology of the retina, both healthy and degenerate, is understood in order to optimize any of these strategies.

Electrical stimulation of the retina dates back to 1755 where Charles LeRoy elicited rudimentary flashes of light, “phosphenes”, by electrical stimulation in the eye of a blind man [1]. Technologies and techniques have advanced greatly over the years with several groups producing electrode arrays that can be implanted on, or near, the retina [2–6]. These aim to stimulate the cells remaining in the retina after photoreceptors are lost in diseases such as retinitis pigmentosa and macular degeneration. Despite the relative success of these projects it is still unknown exactly how electrical stimulation activates retinal neurons to produce perceived visual sensation.

Retinal ganglion cells (RGCs) transmit light information down the optic nerve, and are the cells that must ultimately be activated (directly or indirectly) by a retinal prosthesis to produce visual sensation. Accordingly, many research efforts have focused on assessing the direct activation these cells [7–11]. However, several classes of neuron, pre-synaptic to the ganglion cells, survive photoreceptor degeneration and are possible targets for electrical stimulation. Moreover, some groups have suggested that their devices activate these presynaptic cells [3,12], utilizing the existing circuitry of the retina, but this has only been inferred through indirect measurements. Certainly, it would appear that the most parsimonious way to restore vision would involve exploiting as much of the underlying circuitry of the retina as possible. However, it has long been reported that the cells of the inner nuclear layer (INL) undergo significant anatomical reorganization [13] following photoreceptor cell death. Contrasting with the array of anatomical data on this subject, very little work has been completed examining physiological changes that may, or may not, be associated with these anatomical changes. Membrane oscillations and an increase in spontaneous spike activity in a large percentage of retinal neurons have been reported in degenerate retinae [14–16]. However, Trenholm et al. have recently reported that these oscillations in the *rd/rd* retina, at least in relatively young animals, result solely from the lack of photoreceptor input to the network, rather than due to post-degeneration remodeling [17,18].

Direct external electrical stimulation of neurons is generally accepted to cause spiking through activation of voltage-gated sodium (Na_V_) channels [19–21]. However, retinal neurons, with the exception of RGCs, display a minimal expression of Na_v_-channels in comparison to ‘typical’ spiking neurons. Although it is clear that many amacrine cell types [22–25] and some bipolar cell types [26–28] do express Na_v_ channels, the predominant voltage-activated current that can be recorded in these cells is potassium, through voltage-gated potassium (K_V_) channels [29]. Therefore, it is likely that the response of INL cells to external electrical stimulation will differ greatly to that of the RGCs.

One study recorded the responses of displaced amacrine cells of the rabbit retina (located in the ganglion cell layer) to external electrical stimulation showing synaptically evoked responses [30]. However, this has never been replicated in cells of the INL of the mammalian retina, nor has the origin of these synaptic responses been identified. This is surprising considering the number of studies that attribute RGC synaptic inputs, to the electrical activation of photoreceptors, horizontal, bipolar or amacrine cells [3,12,31]. Maraglit et al. recorded membrane potential changes in bipolar cells of the tiger salamander in response to epiretinal stimulation that could be modulated by specific synaptic blockers [32], suggesting mammalian INL cells would exhibit similar responses.

We recorded electrical responses in cells of the INL in both healthy and degenerate retinal tissue in an effort to build a more complete picture of the mechanism of electrical stimulation of the retina and how this may influence the eventual signal that reaches the visual cortex. We report that electrical stimulation elicits both intrinsic and extrinsic membrane potential changes in the majority of cells recorded. When pre-synaptic inputs were blocked, direct activation of both Na_V_ and K_V_ channels was revealed. Activation of both these channels was balanced so that stimulus intensity altered both the amplitude and polarity of the response. We suggest that these findings be taken into account when designing stimulus strategies for future retinal prostheses.

## Methods

### Animals

Ethics: All procedures involving animals were approved and monitored by the University of Western Sydney Animal Care and Ethic Committee, project number: A8967.

Wild-type C57BL/6J mice were purchased from ARC (Canning Vale, Australia) and C57BL/6J-*Pde6b* ^*to -2J*^/J (*rd/rd*) mice were purchased from The Jackson Laboratory (Bar Harbor, Maine, USA). Both strains were bred on site and only offspring (both male and female) >60 days were used. Animals were maintained under a 12: 12hr light: dark cycle at ~300 lux illumination during the daytime. Retinae were excised, and all recordings taken, during the animal’s subjective day.

### Tissue Preparation

All tissue was prepared under normal laboratory lighting conditions. Animals were euthanized using cervical dislocation, eyes enucleated, cut along the ora serrata, and placed in artificial cerebrospinal fluid (ACSF) containing (mM): NaCl 125, NaHCO_3_ 25, KCl 3, NaH_2_PO_4_ 1.25, CaCl_2_ 2 and MgCl_2_ 1, Glucose 25, at room temperature (RT) within 1 min of euthanasia. The retinal slice preparation procedure was adapted from Arman and Sampath (2010) [33]. Briefly, retinae were isolated from the surrounding eye tissue and placed in ~40°C low-melt agarose (Sigma-Aldrich, Australia) dissolved in HEPES buffered ACSF containing (mM): NaCl 140, HEPES 10, KCl 3, NaH_2_PO_4_ 1.25, CaCl_2_ 2 and MgCl_2_ 1, Glucose 25. The agarose block was then submerged in ice-cold ACSF where it quickly solidified. A cube of agarose around the retina was then cut with a scalpel blade and super-glued onto the cutting stage of a vibrating microtome (7000 smz, Campden instruments). The cutting chamber was filled with ACSF at RT and 200 µm sections were taken from the entire retina (the extreme periphery was excluded). Sections were transferred to a holding bath containing ACSF bubbled with 5% CO_2_/95% O_2_ (carbogen) at 34°C. As needed, sections were then transferred to the recording chamber where they were infused with heated, bubbled ACSF at a rate of 5-6 ml/min. A tissue anchor made of platinum with nylon threads (~500 µm apart) was used to keep the tissue in place. This procedure was not altered for *rd/rd* retinae but viable retinal slices were consistently harder to obtain due to difficulties keeping the degenerate retina adhered to the agarose once sliced.

### Electrophysiology

Whole–cell current and voltage clamp recordings were made on cells of the INL with patch electrodes of resistances 5.0–8.0 MΩ. Electrodes were filled with (mM): 120 KMeSO_4_, 10 KCl, 0.008 CaCl_2_, 0.5 EGTA, 1 MgCl_2_, 10 HEPES, 4 ATP − Na_2_, and 0.5 GTP − Na_3_, adjusted to pH 7.2 with KOH. In every case, morphological identification of the recorded cells was made with epi–fluorescent imaging of Alexa Fluor 488 Hydraz (70 µM, Invitrogen) included in the pipette solution. Cells were broadly classified, based on dendritic stratification [34]. Classification was conducted while the recording electrode was in place as withdrawal of this electrode generally resulted in cell body (and sometimes dendrite) removal from the INL. Horizontal cells were only encountered in the wild-type retinae (n=4) and were excluded so that the data could be compared with that of the degenerate retinae. The series resistance (R_S_) was monitored throughout the experiments and was in the range of 10-30 MΩ. Errors associated with R_S_ in voltage-clamp mode were compensated by 60-80% at 5–7 kHz bandwidth, using R_S_ compensation on a Multiclamp 700B amplifier (Molecular Devices). In current-clamp mode, the bridge was adjusted accordingly. Data were low–pass filtered at 10 kHz at the amplifier output and digitized at 50 kHz on a computer running pClamp 10 (Molecular Devices) connected to a Digidata 1440A data acquisition system (Molecular Devices). A liquid junction potential of 5mV has been corrected for all results.

Pharmacological agents were used in the following concentrations: 0.5 mM CdCl_2_ (Sigma), 30 mM tetraethylammonium chloride (TEA; Sigma) and 0.5 µM Tetrodotoxin (TTX; Tocris).

### Electrical stimulation

The retinal slices were stimulated with a 50 µm diameter circular platinum (Pt) electrode, coated with 25 µm thick Teflon (100 µm total diameter; AM Systems) placed behind the photoreceptor layer (wild-type) or behind the INL (*rd/rd*) in a pseudo- “subretinal” configuration. A large (120 µm x 3 mm) Pt wire, submerged in the extracellular fluid, approximately 1 cm from the retina, was used as the stimulus return. Electrical stimuli consisted of anodic–first, charge–balanced, constant–current, rectangular biphasic pulses of 100 µs per phase, without inter-phase delay. For each cell, stimulation intensity was increased until a clearly measurable response could be repeatedly observed. Stimulation was repeated forty times and traces averaged prior to analysis.

### Analysis

Analysis of electrophysiological traces was completed using Axograph software (Sydney, Australia) and amplitudes and latencies compared using GraphPad Prism (San Diego, USA) software. Responding cells were classified as any cell exhibiting a stimulus locked response to electrical stimulation.

## Results

We sought to record directly from cells of the INL in the mouse retina during subretinal, external electrical stimulation, to assess the influence that these cells may have over the eventual output of the retina. The subretinal configuration was used to mirror electrode placement in our previous *in vitro* studies [7,35] and our *in vivo* studies, which adopted a similar posterior electrode placement in the suprachoroidal space [5].

### Electrical responses can be recorded in cells of the inner nuclear layer

**Figure 1:**
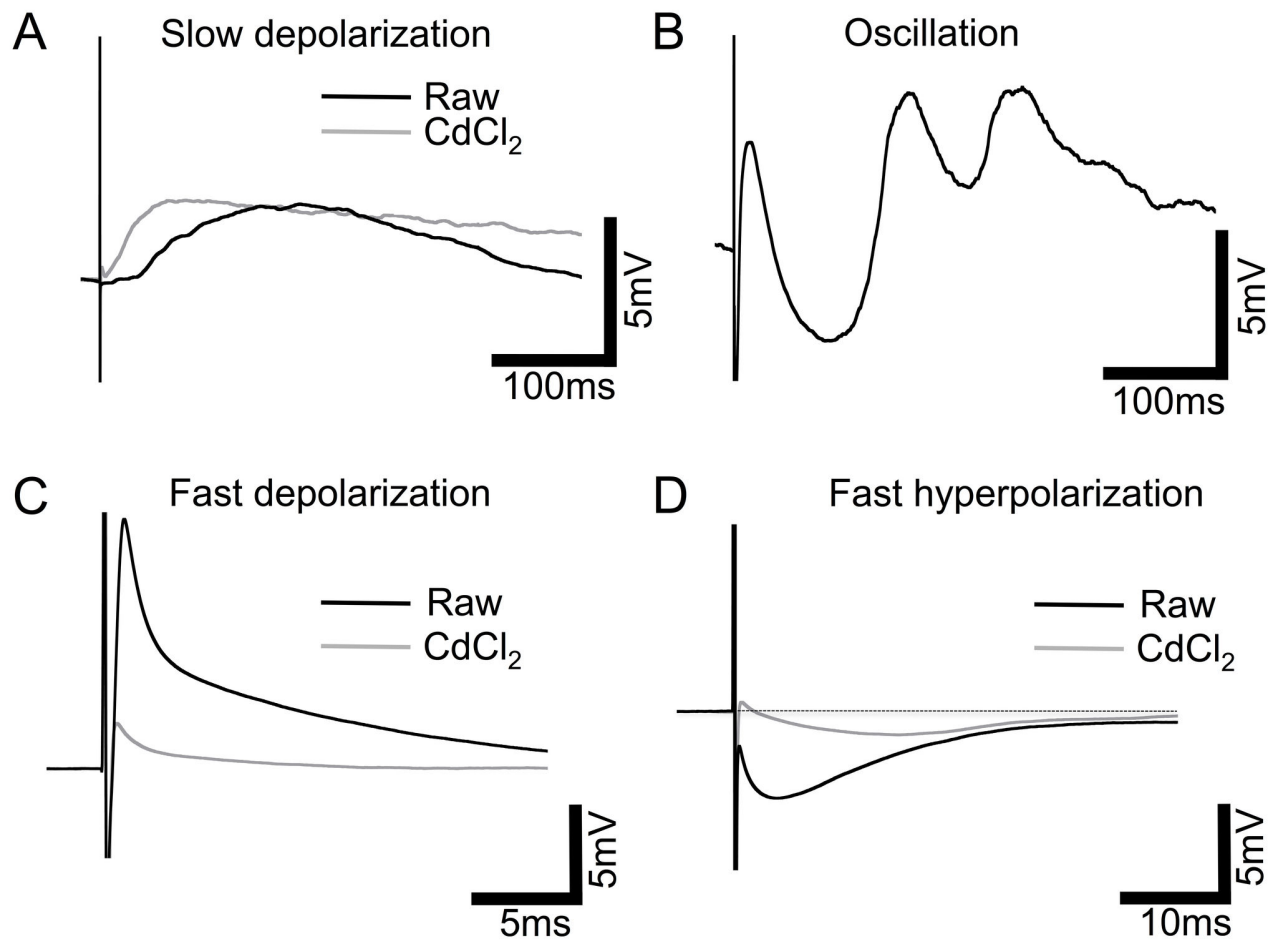
Four patterns of response to electrical stimulation in wild-type retinal INL cells. Representative averaged traces from a variety of INL cell types in the wild-type retina, black traces: A, Slow depolarization classified by the peak of the response occurring >10ms after stimulus onset. B, Oscillation, positive and negative responses continuing >10ms after stimulus onset. C, Fast depolarization (<10ms after stimulus onset). D, Fast hyperpolarization (<10ms after stimulus onset), dotted line depicts baseline. Grey traces: representative averaged traces (not necessarily from the same cell) after treatment with the synaptic blocker CdCl_2_ (0.5mM), response amplitudes were greatly reduced and the oscillation response completely blocked. Note different scales for fast responses.

An array of responses could be elicited from cells of the INL of wild-type mice using external electrical stimulation with ~66% (38/58) of cells responding. Responses were extremely varied from cell to cell, however, four prevalent response patterns could be classified: A) slow depolarization, characterized by the peak occurring >10 ms after stimulation; B) oscillation, positive and negative responses as described previously [30]; C) fast depolarization/spikelet, peak occurring <10 ms after stimulation; and D) fast hyperpolarization, peak occurring <10 ms after stimulation (Figure 1, black traces). Response type, amplitude and latency varied greatly between, and among morphologically identified cell types (Table S1). Additionally, more than one type of response was frequently exhibited by the same cell; specifically, fast hyperpolarization and fast depolarization were often observed together. The amplitude of responses varied with stimulation intensity, therefore, amplitudes have been normalized to stimulation charge density to allow for comparison. Slow depolarization responses averaged 5.6±3.77 mV/mC.cm^-2^ (n=12) with a latency (to peak) of 57.7±14.3 ms. Fast depolarization was exclusively recorded in cells with a measurable transient inward current (determined by an internal voltage step protocol) and responses averaged 3.5±0.84 mV/mC.cm^-2^ (n=8) with a latency of 3.24±1.06 ms. Fast hyperpolarization responses were by far the most prevalent over varying cell types and averaged -2.7±0.98 mV/mC.cm^-2^ (n=17) with a latency of 4.3±0.35 ms. Oscillatory responses were seen in n=6 cells but varied greatly in number of oscillations, polarity, amplitude and latency meaning empirical measurements for comparison were not appropriate.

The variability of this oscillatory response, and the common occurrence of what appeared to be ‘compound’ responses, with two or more response types occurring simultaneously, led us to examine cell responses in the absence of synaptic inputs. We used the Ca^2+^ channel blocker CdCl_2_ (0.5 mM) to eliminate synaptic transmission (although this does not exclude cell: cell communication via gap junctions which are particularly prevalent in the INL). All response types could be observed in the presence of CdCl_2_ with the exception of oscillation (Figure 1, grey traces; Table S2) but amplitudes of the fast responses were significantly reduced (unpaired t-tests, P<0.05). Latencies did not differ significantly. The normalized amplitudes in the presence of CdCl_2_ with slow depolarization responses averaged 0.49±0.27 mV/mC.cm^-2^ (n=3) with a latency (to peak) of 34.9±17.5 ms. Fast depolarization responses averaged 0.42±0.14 mV/mC.cm^-2^ (n=3) with a latency of 1.13±0.37 ms. Fast hyperpolarization responses averaged -0.39±0.08 mV/mC.cm^-2^ (n=5) with a latency of 6.9±2.19 ms. The slow depolarization response, although observed in n=3 cells in the presence of CdCl_2_, was often completely blocked by the addition of CdCl_2_ (n=9). Conversely, the fast response types were never eliminated by synaptic block, although substantially reduced. Taken together, these data indicate that a large component of the electrical response recorded in INL cells originates presynaptically, but the fast responses recorded are likely due to direct activation of the recorded cell.

### Degenerate retinae exhibit INL responses to electrical stimulation

**Figure 2:**
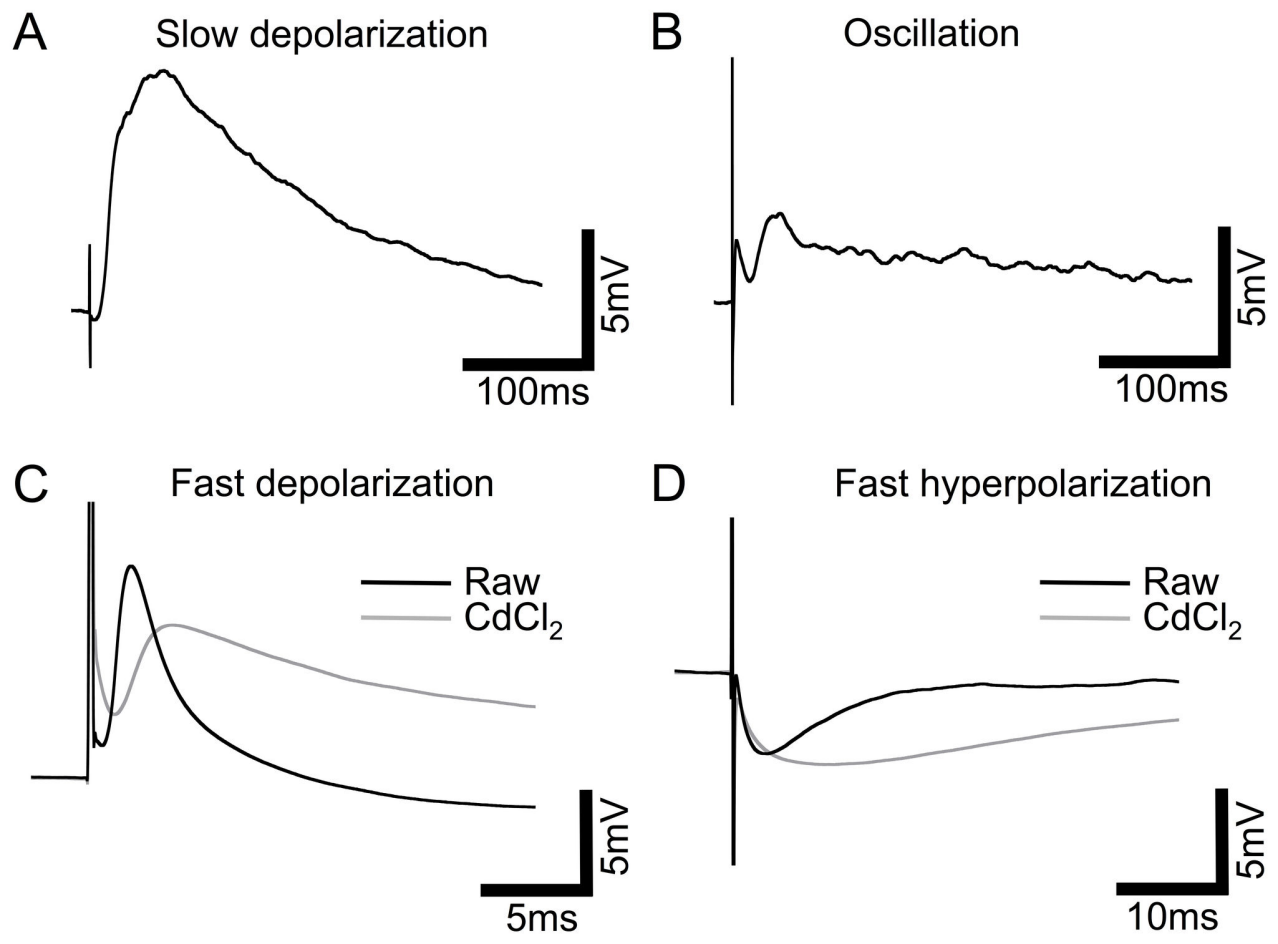
Four patterns of response to electrical stimulation in *rd/rd* retinal INL cells. Representative averaged traces from a variety of INL cell types in the *rd/rd* retina, black traces: A, Slow depolarization (peak occurring >10ms after stimulus onset). B, Oscillation, positive and negative responses continuing >10ms after stimulus onset. C, Fast depolarization (<10ms after stimulus onset). D, Fast hyperpolarization (<10ms after stimulus onset). Grey traces: representative averaged traces (not necessarily from the same cell) after treatment with the synaptic blocker CdCl_2_ (0.5mM), response amplitudes were not significantly altered but slow depolarization and oscillation responses were completely blocked. Note different scales for fast responses.

All four response types observed in the wild-type were also seen in the retinally degenerate *rd/rd* mouse (Figure 2, black traces) with ~82% (23/28) cells responding. However, slow depolarization responses were much less common with only three cells showing this kind of response (13% vs. 32% of cells in the wild-type). Similar to the wild-type, response type, amplitude and latency varied greatly between, and among cell types (Table S3). The response amplitudes and latencies are as follows: slow depolarization responses averaged 5.16±1.98 mV/mC.cm^-2^ (n=3) with a latency of 40.01±11.26 ms. Fast depolarization responses averaged 9.25±3.6 mV/mC.cm^-2^ (n=8) with a latency of 1.7±0.44 ms (again, exclusively recorded in cells with a measurable transient inward current). Fast hyperpolarization responses were, again, the most prevalent over varying cell types and averaged -3.45±0.84 mV/mC.cm^-2^ (n=12) with a latency of 4.6±1.18 ms. Oscillatory responses were seen in n=2 cells but also varied in number of oscillations, polarity, amplitude and latency. Latency, and normalized response amplitude, for the three analyzed response types did not significantly differ between the wild-type and *rd/rd* retinae (unpaired t-test, P>0.05). However, we believe these comparisons should be interpreted cautiously due to variation in slicing protocol, electrode position and lower sample size in the degenerate retinae.

In the presence of CdCl_2_, only fast responses were observed (Figure 2, grey traces; Table S4). Unlike the wild-type retinae, responses were not significantly reduced in amplitude (or latency) by the addition of CdCl_2_ (unpaired t-test P>0.05). Fast depolarization responses averaged 4.76±2.5 mV/mC.cm^-2^ (n=5) with a latency of 2.5±0.68 ms. Fast hyperpolarization responses averaged -1.6±0.67 mV/mC.cm^-2^ (n=4) with a latency of 4.1±1.59 ms. The lack of reduction in the amplitude of the response suggests that the intrinsic response of each cell to electrical stimulation predominately determines the likely output of the cell in the degenerate retina. This is in contrast to the wild-type retinae where responses are greatly influenced by presynaptic potentials.

When response amplitudes and latencies in the presence of CdCl_2_ were compared between wild-type and *rd/rd*s no significant difference could be detected despite the apparent large difference in mean values (unpaired t-test, Welch’s correction, P>0.05).

### Dose response relationship reveals a reversal in response polarity

**Figure 3:**
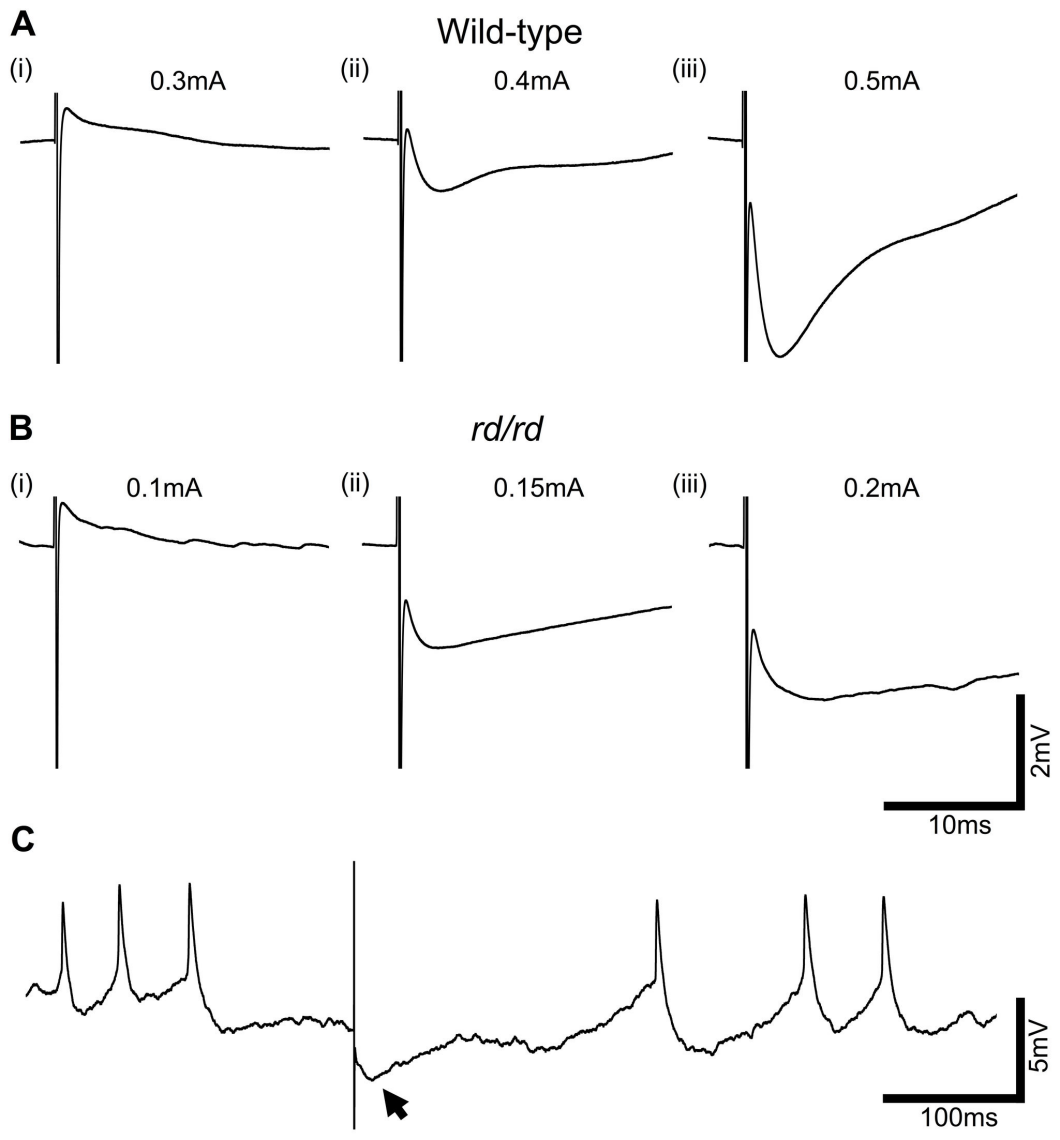
Response amplitude and polarity change in a dose dependent manner with stimulus intensity. A and B, Representative averaged traces from wild-type and *rd/rd* INL cells, under CdCl_2_ (0.5mM), display fast depolarization responses at low stimulus amplitudes that reverse in polarity as stimulus intensity is increased. C, Raw trace from the *rd/rd* cell shows the existence of a large amplitude membrane oscillation, even under synaptic block, that is larger than the electrically evoked response, shown with arrow.

To examine the putative intrinsic responses elicited in the presence of CdCl_2_ in more detail, we assessed the dose response relationship to electrical stimulation. We specifically used cells that exhibited both inward and outward currents when assessed by an internal voltage-step protocol so that both fast depolarization and hyperpolarization responses to electrical stimulation could be assessed. As the charge density of stimulation was increased, the polarity of the response was reversed (wild-type: n=5, Figure 3A; *rd/rd*: n=4, Figure 3B). The fast, depolarizing response seen at lower stimulus intensities was overwhelmed by the fast hyperpolarizing response as the stimulation current was increased. The balance of these two responses varied between cells but seemed to follow the size of inward and outward currents expressed by each specific cell. These data suggest that the fast depolarizing response may be driven by Na_v_ channels, and the fast hyperpolarizing by K_v_ channels.

### Membrane potential oscillation in degenerate INL cells may mask electrical response

Figure 3B traces from *rd/rd* retinae are averaged from repetitive electrical stimuli (40 repetitions), and although they show the same pattern as Figure 3A, it is clear from the baseline that significant background noise remains. Figure 3C shows a raw trace from one of these electrical stimuli. Significant resting oscillation of the membrane potential is evident, even under synaptic block, meaning the electrically elicited fast hyperpolarization response (marked with arrow) was small in comparison. Resting membrane oscillation of varying frequencies and amplitudes was observed in 15/28 cells, in all types of cell recorded except cone OFF bipolar cells, in the *rd/rd* retinae (not apparent in the wild-type retina) and may present a significant problem when electrically exciting these cells due to the small signal to noise ratio.

### Fast responses reflect activation of Na_v_ and K_v_ channels

**Figure 4:**
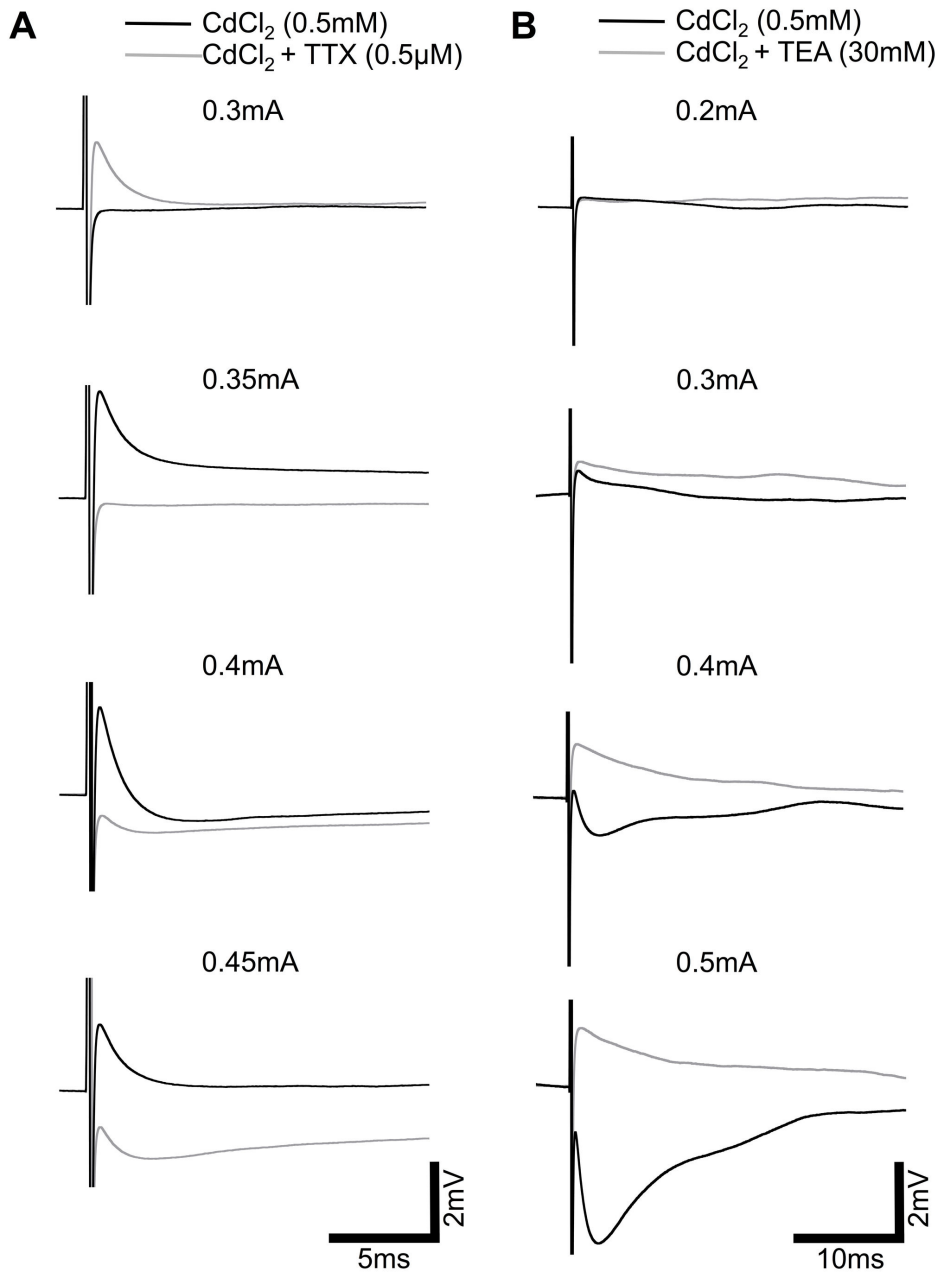
Fast responses originate from direct activation of Na_V_ and K_V_-channels. A, Representative averaged traces of the dose response relationship of a wild-type INL cell under CdCl_2_ (0.5mM), black traces, and subsequently the same cell under CdCl_2_ + TTX (0.5µM), grey traces, show the block of the fast depolarization component. B, Dose response relationship of a wild-type INL cell under CdCl_2_ (0.5mM), black traces, and subsequently the same cell under CdCl_2_ + TEA (30mM), grey traces, shows the block of the fast hyperpolarization component. Scale bar applies to all traces.

The previous data led us to hypothesize that the depolarizing response originates from activation of Na_V_-channels and the hyperpolarizing from K_V_-channels. To further investigate this we used TTX (0.5 µM) to block TTX sensitive Na^+^-channels, and TEA (30 mM) to block TEA-sensitive K^+^-channels. TTX did indeed block the fast depolarizing component (n=5; Figure 4A), while TEA blocked the fast hyperpolarizing component (n=8; Figure 4B) supporting our hypothesis. It is clear from these data that the two responses antagonize each other producing a ‘compound’ response that is dependent on stimulus intensity and voltage-gated channel expression levels. When CdCl_2_, TTX and TEA were applied simultaneously threshold responses were completely abolished (n=4).

**Figure 5:**
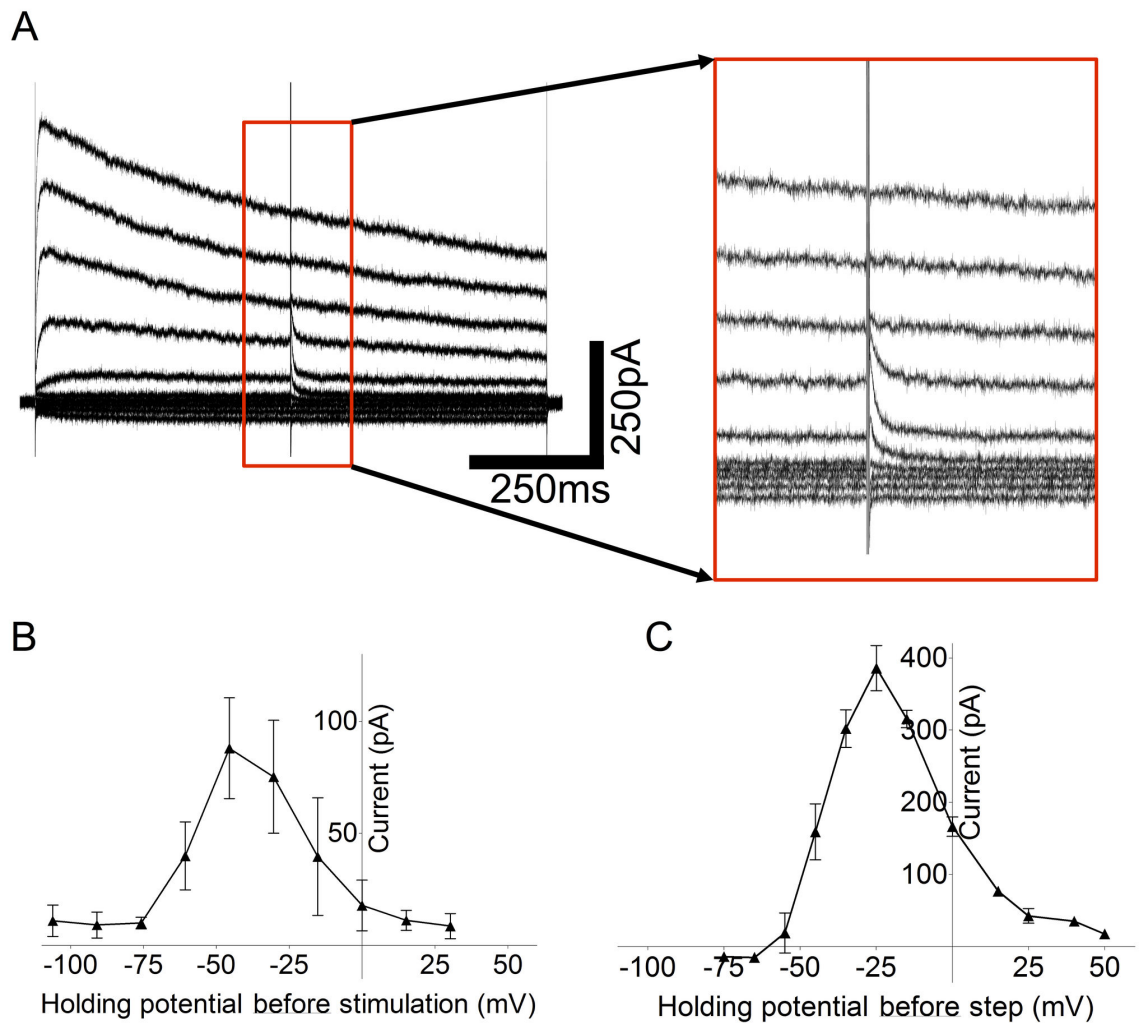
Current-voltage relationship of external electrically evoked responses. A, Raw traces recorded under voltage-clamp conditions stepping the holding potential from -105mV to 30mV in 15mV steps. External electrical stimulation is delivered at t=500ms during the voltage step. Traces show the observed currents immediately following the stimulus artifact, inset box: 200ms, 700pA. B, Current-voltage relationship for n=6 wild-type INL cells displayed an n-shaped response type to external electrical stimulation with a peak ~ 50mV. Response was measured as the difference between the current immediately preceding, and the peak of the current following, the stimulus artifact. C, Current-voltage relationship displayed in B was replicated in n=3 wild-type INL cells using the voltage-clamp by the addition of an 100µs, 100mV internal step, delivered at successive holding potentials, instead of the external electrical stimulation.

Although direct activation of Na_V_ channels is generally accepted to occur during external electrical stimulation [35,36], direct activation of K_V_ channels has never been specifically reported. We therefore analyzed this response in more detail, in cells only exhibiting fast hyperpolarizing responses, by assessing the currents elicited by electrical stimulation using standard voltage-clamp techniques. Membrane potential was stepped to various voltages, from a holding potential of -65 mV, in voltage-clamp mode and the amplitude and reversal potential for the currents elicited immediately following electrical stimulation determined (n=5). Amplitude was calculated as the difference between the current immediately before the stimulus, and the peak current immediately following the stimulus (Figure 5A). We were surprised to observe the IV relationship shown in Figure 5B. Further consideration produced a possible explanation: this protocol would often be used when applying pharmacological agents to examine the reversal potential for the evoked current. However, when electrically stimulating from an external electrode, although we are holding the inside of the cell at a certain voltage with respect to a distant return, the charge in the area immediately surrounding the cell will be altered, changing the *actual* membrane potential of the cell. For this reason, we suggest, the IV curve shows further activation of K_V_-channels above the voltage at which the cell is ‘held’ by the voltage-clamp amplifier. At some point, when the cell is stepped to very depolarized potentials, all available K_V_-channels are already open and a further response cannot be elicited. We sought to replicate this response using only the voltage clamp by adding an additional internal stimulation step to the voltage step protocol to try and mimic the external stimulation. Addition of a brief (100 µs) internal step to further depolarize the cell gave us the same pattern of response (n=3; Figure 5C), suggesting that the IV curve in Figure 5B does indeed reflect an intrinsic K_V_-channel response.

## Discussion

We show that cells of the INL, namely bipolar and amacrine cells, do indeed respond to external electrical stimulation. Although the responses of these cells were extremely varied, it was evident that electrical stimulation elicited responses via both extrinsic and intrinsic cell activation. Extrinsic, pre-synaptically evoked responses were predominately evident in the wild-type retina, suggesting the contribution of rods and/or cones. These data highlight the need for more research on retinally degenerate animal models. A visual prosthesis will most likely be needed to interact with both healthy and degenerate retinal areas, especially in the case of age-related macular degeneration (AMD). The most prudent way of achieving this would be to employ altered stimulus paradigms, rather that different hardware. These data will help inform development of such paradigms.

In the absence of pharmacological blockers, we report that responses from the retinally degenerate *rd/rd* mouse retinae did not differ significantly in response type observed, amplitude or latency, from that of the wild-type. However, the most noticeable difference was the relative under-representation of ‘slow’ (slow depolarization and oscillation) responses in the *rd/rd* retina. These data suggest that the slow responses in wild-types may originate primarily in the outer retina, likely from rods and cones, but possibly horizontal cells which are often lost in retinal degeneration. Freeman et al. (2010) have previously suggested that photoreceptors can be specifically activated using a 5Hz sinusoidal electrical stimulus, although these recordings were not taken directly from photoreceptors [12]. It is therefore likely that the outer retina, which is absent in severe retinal degeneration, may play a large role in the eventual output of this tissue in response to electrical stimulation. More specific pharmacological data is required and/or direct photoreceptor recordings to confirm this, but this is certainly a serious consideration for those testing the efficacy of any such device in a non-retinally degenerate animal model.

The size of the electrically evoked responses recorded in the INL cells in both wild-type and *rd/rd* retinae are comparable to responses to mesopic light previously recorded in bipolar and amacrine cells [24,37,38]. This suggests that these electrically evoked responses likely have a significant influence on the output of RGCs.

Response amplitude and latency for the three analyzed classes of response (slow depolarization, fast depolarization/hyperpolarization) were not significantly different between wild-type and *rd/rd* retinae. This does not agree with published reports from RGCs in *rd/rd* animals that exhibited much higher thresholds for activation when compared to wild-types [39,40]. Again, the limitations of the slice protocol likely accounts for this disparity. The stimulating electrode was positioned very close to the INL cells in the *rd/rd* retina in our protocol (closer than in the wild-type), whereas, in wholemount experiments, glial scaring may cause the electrode to be more distant. Additionally, the above experiments only recorded from RGCs, leaving the possibility open that signals from the INL are not transmitted as effectively in the degenerate retina. This last point is important considering the anatomical rearrangements that have been shown to occur in the degenerate retina following photoreceptor degeneration [13]. It would not be unexpected for synaptic inputs to RGCs from the INL to be fundamentally altered.

The retinal slice protocol is the easiest way to locate, record from, and morphologically classify cells of the INL. However, we do not believe it is best model system for electrical stimulation in an intact mammalian retina. Some synaptic inputs are, of course, removed during the slice process and the orientation of the slice can greatly influence the degree by which this occurs. Most slices were taken from the central retina, but, when a spherical object, such as the retina, is cut in one horizontal plane, the resulting slices will contain slightly different orientations of the radially aligned cells of the retina. It is for this reason that, in this study, we sought to concentrate on characterization of the intrinsically evoked membrane potentials.

Using the Ca^2+^ blocker CdCl_2_ we have removed pre-synaptically evoked responses in INL cells. It is impossible to classify these responses as purely “intrinsic” to the recorded cell due to extensive gap junction coupling and the possible activation of glial cells that may influence neuronal responses via local K^+^ buffering. However, when taking into account the further pharmacology (Figure 4) and the latency of the responses, the evidence suggests that the ‘fast’ responses recorded under CdCl_2_ represent direct activation of the cells. The origin of the slow depolarization response observed in three wild-type cells is unclear, and requires further investigation. While pre-synaptic inputs clearly exert a large influence over the recorded response in cells of the wild-type INL, this influence is less important in the *rd/rd* retinae. It is possible that pre-synaptic inputs in the wild-type retina, from cells of the outer retina, create an underlying excitability in these INL cells that potentiates intrinsic responses when excited with electrical stimuli. The reason for the lack of intrinsic response reduction in the presence of CdCl_2_ in the *rd/rd* INL cells is less clear, but likely reflects connectivity and excitability changes that occur in this layer following photoreceptor degeneration. It is important to note that CdCl_2_ will also block voltage-activated Ca^2+^ channels, so it is possible that a component of the intrinsic response is lost after application of this compound. Further investigation using alternate synaptic blockers would be required to examine this putative response in more detail.

We have presented here, the first indication that K_V_-channels are activated directly by external electrical stimuli. Generally, ‘typical’ spiking neurons express large Na^+^ currents and fire action potentials, however, retinal neurons, with the exception of RGCs, are different in this respect as despite expressing large K^+^-currents, few exhibit large Na^+^-currents. This allows these cells to respond to light with graded potentials, rather than action potentials, thus retaining more light information. The signal is then further processed before digitization to action potentials in the RGCs. Many amacrine cells and some bipolar cells do express Na_v_-channels, and can fire action potentials, but these are generally smaller than a full action potential, or only one spike can be elicited [22,25–28]. It is this property of INL cells that has allowed us to identify simultaneous electrical activation of both Na_v_-channels (as expected from previous studies) and K_V_-channels. Importantly, for these cells, this means a balance between inward and outward currents that dictates the polarity of the eventual response. We have conclusively shown that response polarity can be reversed simply by increasing stimulation intensity. This may have a crucial impact on the stimulation paradigms used in retinal prostheses as turning up the stimulation may not simply increase the signal as previously expected. Furthermore, K_V_-channels should also be activated in spiking neurons, including RGCs, but their influence overwhelmed by the large Na_V_ response in these cells. However, it is important to note the existence of this current, especially when modeling the activation of neurons using external electrical stimuli, an emerging research area.

While examining the putative K_V_-channel response in more detail we utilized a common voltage-clamp protocol, that was used by Margalit et al. (2010) to describe currents elicited by electrical stimulation in INL cells in the salamander retina. The data presented above leads us to propose that this is an unreliable protocol for determination of currents elicited by electrical stimulation. By its very nature, external electrical stimulation alters the membrane potential of the neuron in question, shifting the observed ‘reversal potential’ to an unexpected value. It was only by replicating the effect of external stimulation using transient internal steps, that we were able to decipher the actual change in membrane potential occurring during electrical stimulation, and the likely current that was being activated. More work is required to validate this technique, but it is theoretically possible to accurately replicate the potentials by which the membrane changes during electrical stimulations of varying size, shape and polarity. In this scenario, this technique could be particularly valuable to assess the efficacy of certain external stimulation paradigms on the membrane potential of various neurons.

The biggest difference between the wild-type and the *rd/rd* retina and likely the most important variable to consider when designing a retinal prosthesis, is the existence of a high amplitude resting membrane oscillation in the majority of INL cells which only occurs in the *rd/rd* retina. This resting oscillation has been previously reported to occur in many INL cells and RGCs [14,18] and likely originates from the denervation of gap junction coupled cone ON bipolar cells and AII amacrines [17]. It is clear from Figure 3C that even a relatively large electrically evoked response does not compare to the amplitude of this resting membrane oscillation and the signal may not be detected perceptually due to low signal-to-noise ratio. The nature of this resting membrane oscillation should be investigated further and strategies to dampen the amplitude pursued.

## Supporting Information

Table S1 Response types of wild-type inner nuclear cells. Response types were classified into four types: Fast depolarization classified by the peak of the response occurring <10ms after stimulus onset, fast hyperpolarization (<10ms after stimulus onset), slow depolarization >10ms after stimulus onset and oscillation, positive and negative responses continuing >10ms after stimulus onset. Response types, and number of occurrences are displayed according to morphologically identified cell type which were broadly classified as: rod ON bipolar cell, cone ON bipolar cell, cone OFF bipolar cell, ON stratifying amacrine cell, OFF stratifying amacrine cell, multi-stratifying amacrine cell and narrow stratifying amacrine cell.
(PPTX)

Table S2 Response types of wild-type inner nuclear cells under synaptic block. Response types were classified under synaptic block (CdCl_2_; 0.5mM) into four types: Fast depolarization classified by the peak of the response occurring <10ms after stimulus onset, fast hyperpolarization (<10ms after stimulus onset), slow depolarization >10ms after stimulus onset and oscillation, positive and negative responses continuing >10ms after stimulus onset. Response types, and number of occurrences are displayed according to morphologically identified cell type which were broadly classified as: rod ON bipolar cell, cone ON bipolar cell, cone OFF bipolar cell, ON stratifying amacrine cell, OFF stratifying amacrine cell, multi-stratifying amacrine cell and narrow stratifying amacrine cell.
(PPTX)

Table S3 Response types of *rd/rd* inner nuclear cells. Response types were classified into four types: Fast depolarization classified by the peak of the response occurring <10ms after stimulus onset, fast hyperpolarization (<10ms after stimulus onset), slow depolarization >10ms after stimulus onset and oscillation, positive and negative responses continuing >10ms after stimulus onset. Response types, and number of occurrences are displayed according to morphologically identified cell type which were broadly classified as: rod ON bipolar cell, cone ON bipolar cell, cone OFF bipolar cell, ON stratifying amacrine cell, OFF stratifying amacrine cell, multi-stratifying amacrine cell and narrow stratifying amacrine cell.
(PPTX)

Table S4 Response types of *rd/rd* inner nuclear cells under synaptic block. Response types were classified under synaptic block (CdCl_2_; 0.5mM) into four types: Fast depolarization classified by the peak of the response occurring <10ms after stimulus onset, fast hyperpolarization (<10ms after stimulus onset), slow depolarization >10ms after stimulus onset and oscillation, positive and negative responses continuing >10ms after stimulus onset. Response types, and number of occurrences are displayed according to morphologically identified cell type which were broadly classified as: rod ON bipolar cell, cone ON bipolar cell, cone OFF bipolar cell, ON stratifying amacrine cell, OFF stratifying amacrine cell, multi-stratifying amacrine cell and narrow stratifying amacrine cell.
(PPTX)
