# Supplementary material for: Electrical Stimulation of Inner Retinal Neurons in Wild-Type and Retinally Degenerate (rd/rd) Mice
Source: PLoS One. 2013 Jul 11;8(7):e68882. doi: 10.1371/journal.pone.0068882 (PMC3708954; doi:10.1371/journal.pone.0068882)
Supplement: Table S2 — Response types were classified under synaptic block (CdCl2; 0.5mM) into four types: Fast depolarization classified by the peak of the response occurring <10ms after stimulus onset, fast hyperpolarization (<10ms after stimulus onset), slow depolarization >10ms after stimulus onset and oscillation, positive and negative responses continuing >10ms after stimulus onset. Response types, and number of occurrences are displayed according to morphologically identified cell type which were broadly classified as: rod ON bipolar cell, cone ON bipolar cell, cone OFF bipolar cell, ON stratifying amacrine cell, OFF stratifying amacrine cell, multi-stratifying amacrine cell and narrow stratifying amacrine cell. (PPTX) [file pone.0068882.s002.pptx]

## Slide 1
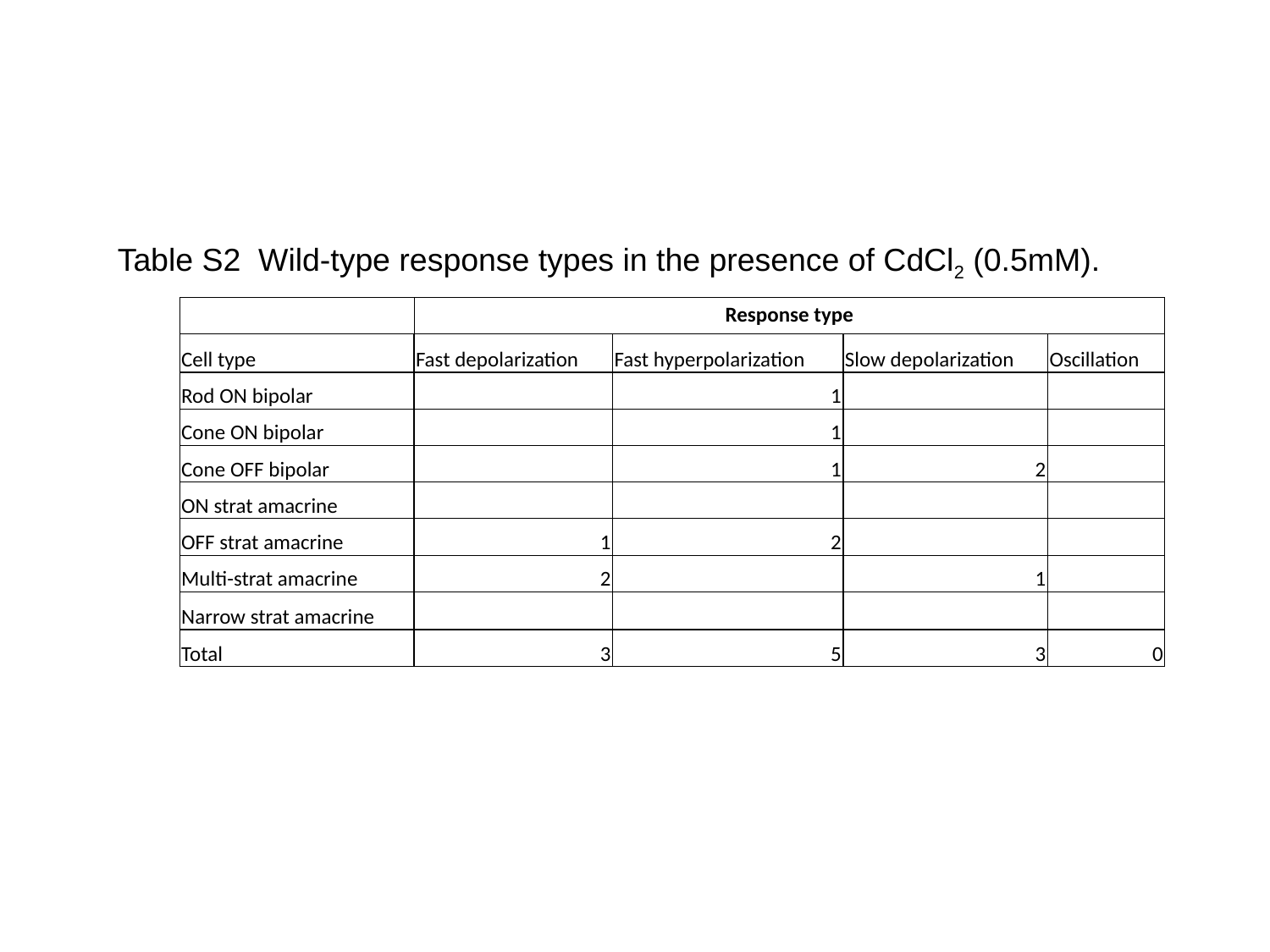

Table S2 Wild-type response types in the presence of CdCl2 (0.5mM).
| | Response type | | | |
| --- | --- | --- | --- | --- |
| Cell type | Fast depolarization | Fast hyperpolarization | Slow depolarization | Oscillation |
| Rod ON bipolar | | 1 | | |
| Cone ON bipolar | | 1 | | |
| Cone OFF bipolar | | 1 | 2 | |
| ON strat amacrine | | | | |
| OFF strat amacrine | 1 | 2 | | |
| Multi-strat amacrine | 2 | | 1 | |
| Narrow strat amacrine | | | | |
| Total | 3 | 5 | 3 | 0 |
